# BOLDconnectR: An R package for streamlined retrieval, transformation, and analysis of DNA barcode data on BOLD

**DOI:** 10.1371/journal.pone.0355496

**Published:** 2026-08-06

**Authors:** Sameer M. Padhye, Liliana Ballesteros-Mejia, Josh Agda, Jireh Agda, Paul D. N. Hebert, Sujeevan Ratnasingham

**Affiliations:** Centre for Biodiversity Genomics, University of Guelph, Guelph, Ontario, Canada; University of Padova: Universita degli Studi di Padova, ITALY

## Abstract

DNA barcode data are essential infrastructure for biodiversity science as they enable scalable species identification and integrative analyses that link sequences to specimens, taxonomy, and geography. The Barcode of Life Data System (BOLD) is a centralized bioinformatics workbench that supports the full barcode data lifecycle: including acquisition, storage, validation, analysis, and dataset publication within a secure collaboration model. While BOLD’s web interface is optimized for interactive dataset assembly and publication, many researchers require programmatic access to both public data and permissioned private records to build reproducible pipelines for curation and analysis prior to release. We introduce BOLDconnectR, an R package that provides authenticated access to BOLD, returns records in the Barcode Core Data Model (BCDM), performs automated transformation into commonly used R data structures, and enables customizable analytic workflows that generate publication-ready outputs.

## Introduction

Curated biological databases are critical to research because they consolidate observations, provide consistent data structures, and make large bodies of evidence computable [1]. In biodiversity science, the value of such infrastructure is especially clear: DNA barcode records are most informative when sequences are connected to specimen metadata, taxonomic interpretation, and collection context, allowing results to be revisited as names change, reference libraries expand, and sampling gaps are filled. DNA barcoding has accordingly become a practical, scalable method for specimen identification and biodiversity monitoring with applications that now extend across ecological networks, conservation, and broad-scale assessments [2,3].

The Barcode of Life Data Systems (BOLD) was built to support this lifecycle. Beyond being a repository, BOLD functions as a web workbench for assembling, validating, analyzing, and publishing DNA barcode data under controlled access [4]. It enables collaborative data assembly and curation within permissioned projects and includes publication workflows that create the durable datasets associated with citable DOIs. Critically, much of the scientific work occurs before release: records are iteratively cleaned, reconciled, and reviewed while they remain private within a project before being packaged into datasets suitable for publication.

While BOLD provides API access, most existing client tools such as ‘bold’ and ‘BOLDigger’ are designed for accessing and working with public data; they lack built-in support for authenticated access to private, pre-publication data into reproducible analytical workflows [5,6]. This limits a common need in barcode research: the ability to run scripted, scalable, organization-specific pipelines for curation and analysis prior to dataset publication. Such pipelines are increasingly important for teams and research networks that must harmonize metadata across contributors and labs, perform targeted diagnostics, and generate publication-ready summaries/exports that accompany dataset release and aid manuscript preparation [7,8].

Here we present BOLDconnectR, an R package that provides access to public records and authenticated, permission-respecting access to private records. BOLDconnectR retrieves records in a structured format based on BOLD’s core data model (https://github.com/boldsystems-central/BCDM) and performs automated transformation into R objects for sequence, spatial, and biodiversity analyses. By doing so, it extends BOLD’s interactive workbench into the R ecosystem, enabling custom pipelines.

## Implementation and overview

BOLDconnectR is implemented in R (≥4.0) and distributed through CRAN and GitHub (https://github.com/boldsystems-central/BOLDconnectR). The package is intentionally compact, focusing on a small number of operations that recur across barcode projects: selecting candidate records, retrieving them, transforming them into analysis-ready objects, and exporting results for reuse. This design reflects the practical lifecycle in BOLD.

Access to authorized private records is enabled through token-based authentication while public records remain accessible without authentication. Tokens are generated by users through their BOLD profile and are designed to rotate periodically. Consequently, scripts should be written with plans to refresh tokens rather than embed long-lived credentials, reducing the risk of inadvertent dissemination through shared code, notebooks, or logs. Users incorporate a current token via *bold.apikey(),* after which retrieval and search functions operate on both public data and private records within the user’s authorization scope.

**Fig 1:**
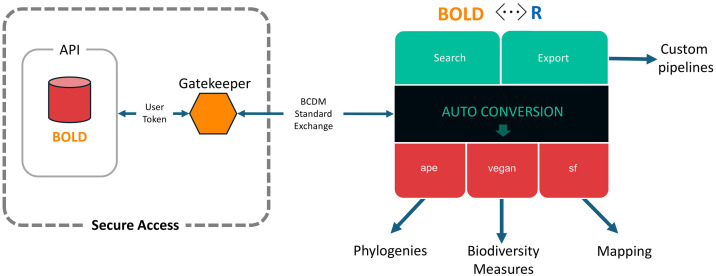
High-level overview of the functionality of BOLDconnectR. The package retrieves data in the BCDM format via token-authenticated APIs (https://boldsystems.org/data/api/), automatically formatting it for downstream analysis. Users can also export datasets in different formats to use within custom pipelines or third-party analytical workflows.

Retrieved records are returned in the Barcode Core Data Model (BCDM), preserving the links between sequences and the specimen metadata, taxonomy, identifiers, and geography. From this representation, BOLDconnectR performs transformation to the input structures required for downstream analyses in R (Fig 1 ‘Auto Conversion’). Importantly, the package provides translations to three widely used analytical ecosystems: ape [9] (sequence handling and phylogenetic workflows), vegan [10] (biodiversity measures and community summaries), and sf [11] (spatial representations and mapping). These packages are among the most broadly adopted tools for analysis of DNA and biodiversity data in R, and BOLDconnectR facilitates their use by converting BOLD records into the specific object classes required by each analysis step based on user-provided parameters.

Functions are organized into four groups. Search functions enable the selection of candidate records based on taxonomy, geography, institution, marker, and collection date, returning identifiers for subsequent record retrieval, with bold.public.search (using the portal API) targeting publicly available data and bold.full.search (using the Data API search) extending searches to include both public and authorized private records (Fig 1 ‘Search’). Retrieval functions download complete records based on identifiers, BINs, or dataset/project codes, with optional filters to refine the returned set (using Data API retrieve). The download function makes requests in batches to avoid straining BOLD servers. After downloading, analysis helper functions provide sequence alignment, distance calculations, biodiversity measures, and mapping via calls to ape, vegan, and sf (and associated dependencies) (Fig 1 ‘Phylogenies’, ’Biodiversity Measures’, ‘Mapping’). Export functions write BCDM and derived products to standard formats (CSV/TSV/FASTA), including parameters to customize FASTA headers assembled from selected record fields (Fig 1 ‘Export’).

BOLDconnectR has been evaluated against two public BOLD datasets spanning approximately 10^3^ (n = 847; DS-LONO2 – [12]) – 10⁴ (n = 9087; DS-SPHD2401*-* [13]) records. The package is under active development, and future work will aim to extend its functionality to support additional workflows as BOLD evolves and as community needs emerge. Planned extensions will emphasize publication oriented pipeline components that support dataset preparation while enabling analyses on private data.

### Use cases

BOLDconnectR supports reproducible, scriptable workflows over public and user-authorized private BOLD records during dataset preparation and publication [4].

#### Use case 1: Permissioned retrieval of pre-publication records.

Private access is enabled with a token generated in the user’s BOLD profile (tokens rotate periodically. *bold.apikey()* saves the token in the R environment for the length of the R session. Retrieval is performed with *bold.fetch(),* where the *get_by* parameter specifies the identifier type and identifiers provide one or more values (e.g., dataset codes, BINs, or process IDs).

*bold.apikey(“BOLD_TOKEN”)*

*bcdm <- bold.fetch(get_by = “dataset_codes”, identifiers = “DS-SPHD2401”)*

Optionally, filters can be applied during retrieval to narrow the result set by common facets such as taxonomy or geography which is useful during iterative curation.

*bcdm_sub < - bold.fetch(get_by = “dataset_codes”, identifiers = “DS-SPHD2401”, filt_taxonomy = “Megaselia altifrons”, filt_geography = “Lkr. Rems-Murr”)*

#### Use case 2: Search then retrieve a targeted subset.

When defining a study set, a two-step pattern is often preferable: search broadly to obtain identifiers, then retrieve full records only for those identifiers. In *bold.full.search()*, parameters such as taxonomy and geography can be supplied as lists; the result contains process IDs that can be passed directly to *bold.fetch()*. The *bold.public.search()* can also be used instead if dealing with public only records.

*bold.apikey(“BOLD_TOKEN”)*

*hits < - bold.full.search(taxonomy = list(“Panthera leo”), geography = list(“India”))*

*bcdm <- bold.fetch(get_by = “processid”, identifiers = hits$processid)*

#### Use case 3: Analysis-ready conversion and publication-oriented export.

BOLDconnectR reduces friction for downstream analysis by converting BCDM to formats used by widely adopted R ecosystems (ape for sequence/phylogenetic workflows, vegan for biodiversity summaries, and sf for mapping) followed by the analysis. For example, with biodiversity metric generation, users control analysis intent via parameters such as *taxon_rank used* (e.g., species, genus), *site_type* (how samples are grouped), *location_type* (the geographic resolution), and *diversity_profile* (e.g., richness). When executed, the function converts the BCDM data into a vegan occurrence matrix, analyses it, returning results.

*bold.apikey(“BOLD_TOKEN”)*

*bcdm <- bold.fetch(get_by = “dataset_codes”, identifiers = “DS-LONO2”)*

*div < - bold.analyze.diversity(bcdm, taxon_rank = “species”, site_type = “locations”, location_type = “region”, diversity_profile = “richness”)*

For publication-oriented outputs and downstream interoperability, *bold.export()* can write sequence data in FASTA format with customized headers built from selected BCDM fields (cols_for_fas_names), allowing traceable identifiers to aid phylogenetic or curation workflows.

*bold.export(bcdm, export_type = “fas”, export = “lono2.fas”, cols_for_fas_names = c(“bin_uri”, “species”, “country.ocean”))*

## Conclusion

BOLD provides a secure, collaborative workbench for assembling, curating, analyzing, and publishing DNA barcode data within private projects. BOLDconnectR complements this environment by enabling permissioned programmatic access to BOLD records in R while preserving structured links among sequences, specimens, taxonomy, and geography. By automating conversion to formats used by widely adopted analytical tools it reduces data handling overhead. The package bridges an important gap by providing the first programmatic interface to access and analyze both public and permissioned Barcode data on BOLD. The package is under active development, with the aim of expanding support for additional workflows and interoperability as BOLD and community needs evolve.
